# A Randomized Case Series Approach to Testing Efficacy of Interventions for Minimally Verbal Autistic Children

**DOI:** 10.3389/fpsyg.2021.621920

**Published:** 2021-05-24

**Authors:** Jo Saul, Courtenay Norbury

**Affiliations:** ^1^Department of Language and Cognition, University College London, London, United Kingdom; ^2^Department of Special Needs Education, University of Oslo, Oslo, Norway

**Keywords:** autism, minimally verbal, intervention, randomization, speech, parent-mediated, single case design

## Abstract

**Background:**

Randomized Controlled Trials (RCTs) are the gold standard for assessing whether an intervention is effective; however, they require large sample sizes in order to detect small effects. For rare or complex populations, we advocate a case series approach as a more realistic and useful first step for intervention evaluation. We consider the importance of randomization to such designs, and advocate for the use of Randomization Tests and Between Case Effect Sizes to provide a robust and statistically powerful evaluation of outcomes. In this tutorial, we describe the method, procedures, and analysis code necessary to conduct robust single case series, using an empirical example with minimally verbal autistic children.

**Method:**

We applied a pre-registered (https://osf.io/9gvbs) randomized baseline design with between-case effect size to a case series (*n* = 19), to test the efficacy of a novel, parent-mediated, app-based speech production intervention (BabbleBooster) for minimally verbal autistic children. Parent-rated probe scores were used to densely sample performance accuracy over time.

**Results:**

Parents were able to reliably code their children’s speech productions using BabbleBooster. A non-significant Randomization Test and small Between-Case Effect Size (*d* = 0.267), suggested there was no evidence that BabbleBooster improved speech production in minimally verbal autistic children, relative to baseline scores, during this brief period of intervention.

**Conclusion:**

The current analyses exemplify a more robust approach to examining treatment effects in rare or complex populations, where RCT may be difficult or premature to implement. To facilitate adoption of this method by researchers and practitioners, we provide analysis code that can be adapted using open source R packages. Future studies could use this case series design to evaluate interventions aiming to improve speech and language outcomes for minimally verbal autistic children, and other heterogeneous and hard to reach populations.

## Introduction

The core characteristics associated with autism are differences in social engagement and behavioral rigidity (American Psychiatric Association, 2013). Expressive and receptive language trajectories are highly heterogeneous, with an estimated 25% of autistic individuals^1^ remaining minimally verbal beyond school age, indicating few or no words are spoken on a regular basis (Lord et al., 2004; Norrelgen et al., 2014). Development of speech by age five is one of the strongest predictors of functional outcome (e.g., academic qualification, paid employment, independent living, mental health) in adulthood (Szatmari et al., 2003; Howlin, 2005), yet a recent Cochrane review highlighted the paucity of robustly designed and adequately powered studies of language interventions for minimally verbal autistic participants (Brignell et al., 2018). High quality intervention studies are thus urgently required, yet the financial and logistical challenges of recruiting and testing a large sample of minimally verbal autistic participants can be prohibitive. The current study describes and illustrates the use of an alternative study design suitable for smaller heterogeneous samples: the randomized case series. We use data collected in a pilot study of a parent-mediated app-based speech production intervention, developed specifically for minimally verbal autistic children, to illustrate appropriate design and analysis techniques.

The Randomized Controlled Trial (RCT), in which a large group of participants is randomly allocated either to receive the treatment or a control condition, is considered the gold standard method with which to evaluate the efficacy of intervention trials (Sibbald and Roland, 1998; Kendall, 2003). Despite widespread adoption of RCTs with neurodevelopmental conditions, certain circumstances can make implementing an RCT difficult: the target population may be rare, difficult to recruit in sufficient numbers, and/or extremely heterogeneous (e.g., individual targets may need to vary by participant). RCTs are also costly to implement, and thus only appropriate once an advanced stage of intervention development has been reached, following the incorporation of prior rounds of piloting and feedback (Craig et al., 2006).

An additional pitfall of any between-subject design such as RCTs, is their reliance on single time-point measurements of pre- and post-intervention performance. This requires the comparison of the same outcome, measured on only two occasions. In an emerging skill, or for a population with highly variable test performance due to attentional or behavioral factors, this method risks over- or underestimating a treatment effect. The assumption that grouping participants at random will ‘equal out’ this measurement error may only be true in participants with a homogenous profile, which is rarely the case in neurodevelopmental conditions. Dense sampling, in which there is repeated assessment of the outcome measure both before and during the intervention, provides a more robust measurement method in populations with high heterogeneity or where individual differences are of special interest (Wilson, 2011).

A viable alternative to the RCT is the Single Case Experimental Design (Kazdin, 2019), in which each participant serves as their own control and multiple measurements are taken across at least two experimental phases, usually baseline and intervention. The overall goal is to establish a functional relationship between the intervention and a change in the dependent variable of interest. Single Case Experimental Designs come in many formats, predominantly either a phase design, where baseline and intervention measurement occasions are grouped together in sequential blocks, or an alternating design, where intervention and baseline sessions are interspersed. Features of the intervention usually guide design choice: alternating designs are best suited to interventions that work only while they are ongoing and do not have a lasting effect (e.g., tick chart for target behavior in class), whereas phase designs suit interventions where skills are built up and are expected to be retained over time.

Randomization is a cornerstone of good experimental design as it reduces extraneous confounds and increases internal validity (Barton, 2006). Single Case Experimental Designs can also incorporate randomization, for example in stimuli selection. Howard et al. (2015) advocate for the use of large stimuli sets whereby items are matched for baseline performance and randomly allocated to treatment or control conditions. The quantity of items and their randomized allocation counteracts the problem of regression to the mean, which can lead to spurious treatment effects. This is especially problematic when test performance is highly variable. This design suits word learning studies where there is a large bank of items to draw from, and works for populations that can sustain regular lengthy probes. However, minimally verbal autistic children can rarely attend for long enough to complete large sets of trials, and with speech sound learning there is only a limited number of appropriate targets to incorporate, so this approach does not suit all populations or interventions.

Single Case Experimental Designs are a widely accepted source of evidence in a number of fields such as education (Shadish et al., 2015), medicine (Vohra, 2016), and psychology (Kazdin, 2019). Despite the advantages of being low-cost, easy to implement and extremely flexible, Single Case Experimental Designs have been historically viewed as methodologically inferior (Concato et al., 2000). One reason for this is the lack of statistical tests available to evaluate their results, since they violate parametric assumptions of independence of observations and random sampling from the normal distribution. Single Case Experimental Designs were traditionally analyzed by visual inspection alone, in which observations of the outcome variable are graphed over time and aspects such as level, trend and variability are compared between experimental conditions. This approach incorporates the richness of the data whilst remaining simple and accessible (Heyvaert et al., 2015). However, the lack of objective decision-making guidelines leaves this approach vulnerable to bias and inconsistency between researchers (Matyas and Greenwood, 1990; Parsonson and Baer, 1992; Ninci et al., 2015).

There has been a renewed interest in Single Case Experimental Designs, based on numerous innovative quantitative approaches to their analysis, which go beyond visual inspection (Manolov and Moeyaert, 2017). New methods enable researchers to use Single Case Experimental Designs to robustly test functional relationships between interventions and outcomes, and to compute effect sizes for cross-study comparison and inclusion in meta-analyses. A growing recognition of the value of Single Case Experimental Design when these analytic approaches are incorporated, has led to the establishment of new standards (Shamseer et al., 2015; Tate et al., 2016; Vohra et al., 2016). Replication of effects is crucial (Horner et al., 2005; Kratochwill et al., 2010), and can be achieved in various ways. For instance, using a single participant with three different exposures to or withdrawals of an intervention (ABAB design), or using three participants who each begin an AB phase intervention at staggered start time-points (multiple baseline design). In a multiple base line design, replication of the treatment effect across different individuals who begin the intervention at different times, is a source of internal validity.

An array of books, special journal issues, tutorials and simulations have been published in the past decade, all proffering new ways to statistically analyze Single Case Experimental Designs (see summary in Manolov and Moeyaert, 2017), with no clear consensus on a single standard approach. Furthermore, despite the heavy output of methods papers, published studies employing any of these methods are still rare. The randomization test (described below) is one innovative approach that has been employed in several Single Case Experimental Designs (Wenman et al., 2003; Schulte and Walach, 2006; Hoogeboom et al., 2012; Hwang et al., 2018; Alfonsson et al., 2019; Calet et al., 2019). In addition, the between-case standardized effect size (described below) has recently been used in meta-analysis (Barton et al., 2017). To our knowledge, a practical application that combines these methods has not yet been carried out to evaluate interventions in autistic populations.

Systematic reviews of language interventions in autism incorporating Single Case Experimental Design evidence have either been unable to generate an effect size at all (Lane et al., 2016; Mulhern et al., 2017), or have used the Percentage of Non-overlap statistic (Kane et al., 2010), which is unfortunately limited due to ceiling effects (Parker et al., 2011) and is confounded with length of baseline period (Allison and Gorman, 1993). Furthermore, Lane et al. (2016) assessed naturalistic spoken language interventions in autism for methodological quality and found that only half the Single Case Experimental Design studies (24 studies, *n* = 45) were of adequate quality. In summary, robust analysis measures and quality standards are still sorely lacking in the Single Case Experimental Designs describing language interventions in autism, limiting progress in research, policy, and practice.

The goal of this paper is to demonstrate a practical application of two innovative approaches to statistical analysis of Single Case Experimental Designs: (1) the randomization test, and its subsequent pooling across participants, and (2) a standardized Between-Case Effect Size (BCES), accounting for between-participant variance. These metrics are complementary to and independent of one another. We will briefly describe them, explain why they were chosen rather than potential alternatives, and address common criticisms. An in-depth mathematical and theoretical explanation of why these methods are appropriate can be found in Shadish et al. (2014a, b) and Hooton (1991).

### The Randomization Test

An important way that randomization can be incorporated into Single Case Experimental Designs is by employing randomized assignment and testing functional relationships via the Randomization Test devised by Fischer (Rvachew and Matthews, 2017). This is done by randomly selecting the intervention schedule for a given Single Case Experimental Design from a pre-determined number of permissible schedules. The scope of this random assignment varies by Single Case Experimental Design type: in an alternating design, intervention allocation can be completely randomized (e.g., producing the sequence ABBABABBBBAABA, where A = baseline measurement occasion and B = intervention measurement occasion), whereas in a phase design the baseline and intervention measurement occasions must be grouped together in phases (e.g., AAAAAABBBBBBBBB). The number of permutations from which the allocated schedule is chosen will vary by design type, number of measurement occasions and any further constraints (e.g., a minimum baseline period before intervention is introduced in a phase design).

**FIGURE 1:**
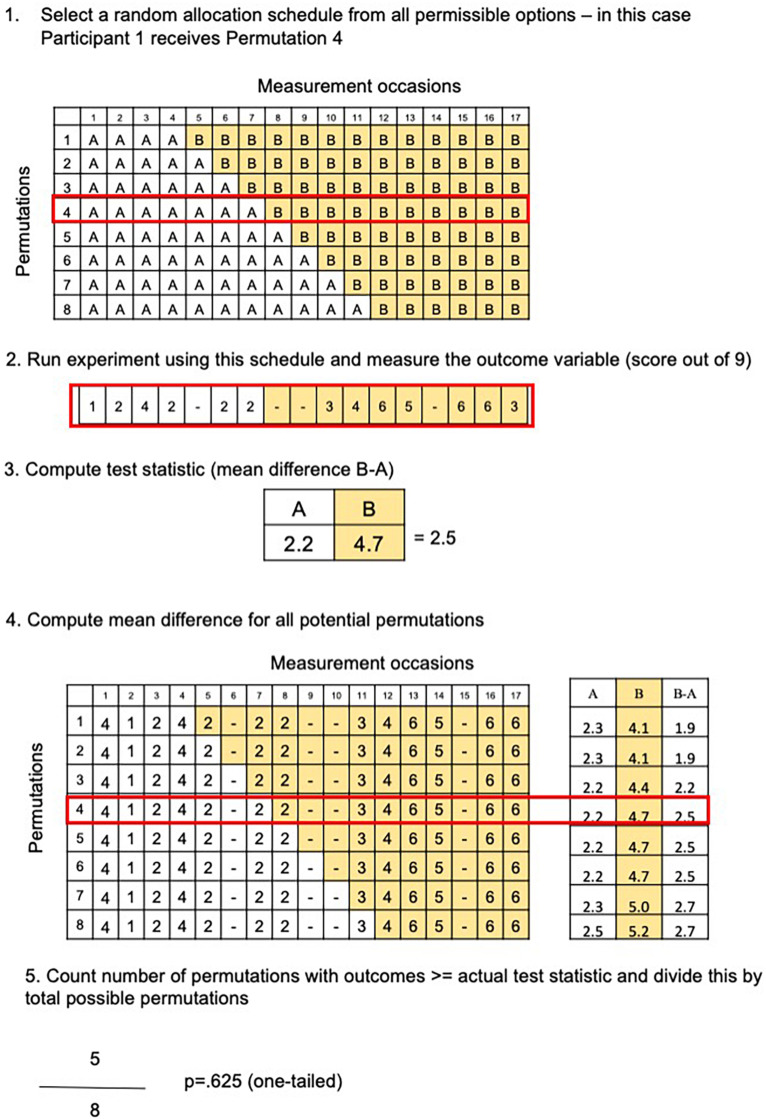
Steps needed to calculate a Randomization Test. **(1)** Random selection of intervention schedule; **(2)** repeated measurement of outcome variable; **(3)** calculation of mean difference between intervention and baseline scores; **(4)** compute all potential mean differences (one for each permissible intervention schedule); **(5)** compare the actual mean difference with all possible outcomes to obtain a rank, e.g., the fifth greatest mean difference out of eight possibilities, which corresponds with a *p*-value of 5/8 or 0.625.

**FIGURE 2:**
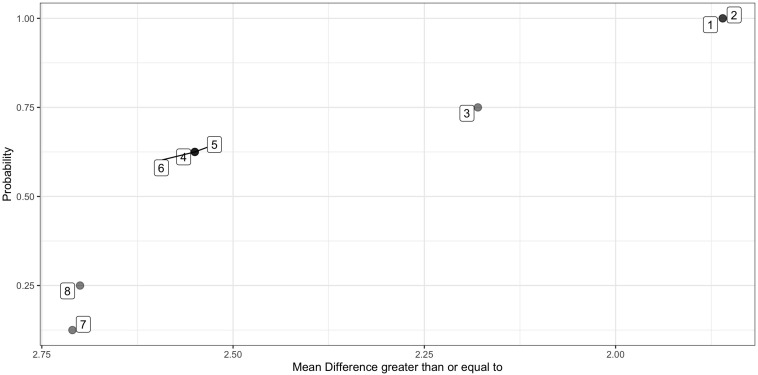
Probability distribution of all possible mean differences. Plots the mean difference for each of eight permissible permutations in rank order, against the likelihood of the mean difference being at least as great, e.g., all mean differences are greater than 1.86, *p* = 1 that any of the eight selected at random will be at least 1.86. Only 1 is greater than or equal to 2.71, therefore the associated if the actual observed mean difference was 2.71 is *p* = 1/8 or 0.125. Data points are labeled according to the permutation number.

**FIGURE 3:**
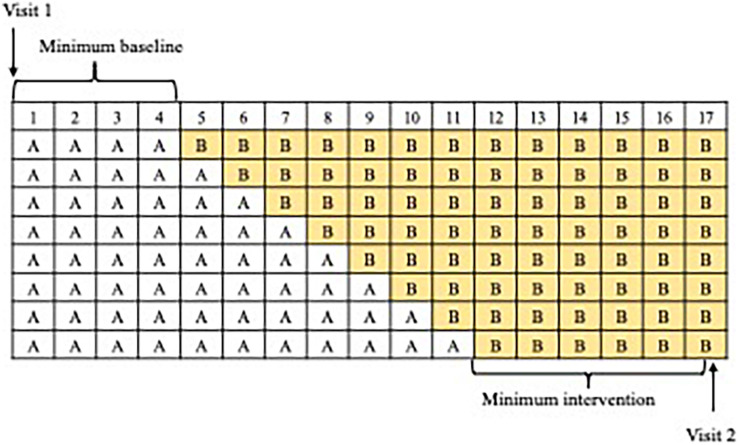
All possible permutations of baseline (A) and intervention (B) weeks.

So long as the intervention schedule was randomly allocated from a number of possible permutations, a Randomization Test can be performed by computing a test statistic (e.g., the mean difference score of A versus B occasions) for each permissible permutation, via resampling. We provide an example using data from the BabbleBooster pilot project in Figures 1, 2 (note that raw scores are used rather than percentages). There are eight possible permutations of the intervention schedule, with a minimum of six and a maximum 13-week treatment period as illustrated in Figure 3). Each schedule includes 17 opportunities to assess the outcome measure; average accuracy during the baseline period (all the A weeks) is then subtracted from average performance during the treatment period (B weeks). We then generate the range of all eight possible mean difference scores (assuming the intervention had started at session 5, 6, 7, 8, 9, 10, 11, or 12) and compare them in size to the actual mean difference obtained. If the intervention had no effect (the null hypothesis), there would be a 1/8 chance that the obtained mean difference would be the greatest score when compared to each and all of the seven other outcomes. The relative ranking of the actual mean difference is thus translated into a *p*-value, for example, if there are eight possible comparisons, and there are five hypothetical outcomes with the same or greater mean difference, this equates to a *p*-value of 5/8 or 0.625.

Conceptually, random assignment strengthens internal validity by counteracting the threats of maturation and history (Heyvaert et al., 2015). The Randomization Test is not linked to a specific test statistic, so if the mean difference is not appropriate, there is flexibility to use a different metric. As a non-parametric test, the Randomization Test is robust to violations of certain assumptions that are difficult to meet in Single Case Experimental Design research, namely independence of observations and random sampling from a normal distribution (Hooton, 1991). Single Case Experimental Design observations usually have a degree of serial dependency, or autocorrelation, and can display trends (Solomon, 2014); the Randomization Test can accommodate linear trend better than a group design (Michiels and Onghena, 2019).

Despite these advantages, randomization remains rare in Single Case Experimental Designs (Heyvaert et al., 2015). One criticism is that the Randomization Test’s power to detect an effect diminishes in the presence of certain non-linear trends such as a delayed intervention effect, a learning curve or an extinction burst (Sierra et al., 2005; Wilson, 2011; Levin et al., 2017). Another issue is that random-assignment of intervention start point is not always possible or desirable. The pre-determined introduction point of an intervention is at odds with response-guided experimentation (Kazdin, 1980), and can be challenging if it is not known how long a stable baseline will take to achieve. Rvachew and Matthews (2017) also highlight the ethical dilemma of potentially giving some participants a very long baseline with many repeated measurement obligations prior to receiving the intervention. However, each participant does receive some exposure to both conditions, unlike an RCT where participants may be assigned to the control group and not receive any of the intervention.

As is evident from the example in Figure 1, if there are only eight possible permutations for a given participant, the lowest achievable *p*-value for a Single Case Experimental Design is 0.125, or 1/8, assuming a one-tailed analysis. A single AB phase Single Case Experimental Design alone is unlikely to have adequate power to detect small improvements in the target measure (Haardörfer and Gagné, 2010; Michiels and Onghena, 2019). Ways to increase power include increasing the number of measurement occasions, or replicating the result by pooling results across participants. *P*-values derived from individual Randomization Tests can be pooled across participants in a case series or multiple baseline design, to determine the likelihood of these p-values occurring by chance, using Stouffer’s Z statistic (Rvachew and Matthews, 2017).

### The Between-Case Effect Size (BCES)

The Randomization Test assesses the significance of a functional relationship between the intervention and a change in the outcome variable, but does not inform us as to the magnitude or variability of this effect. Effect sizes not only convey this important information, but due to their standardization, enable the comparison of effects across studies. Effect sizes are increasingly considered to be more important than *p*-values for interpreting intervention results and informing evidenced-based practice (Wilkinson and Task Force on Statistical Inference, 1999). RCTs have an established standardized effect size, Cohen’s *d* (Cohen, 1977), which can be adjusted to Hedges *g* (Hedges, 1981) for small samples. The unit of comparison is standard deviations of outcome variable. Effect sizes historically developed for Single Case Experimental Designs cannot be standardized in the same way and do not account for between participant variance, in the way that Cohen’s *d* does in a group study (see Odom et al., 2018 for a summary of previous approaches and their limitations). The importance of determining a robust effect size for Single Case Experimental Designs is increasingly recognized (Shadish et al., 2014a), as few studies currently report effect sizes or their variances (Jamshidi et al., 2018).

Many effect size metrics have been proposed for single case experiments (Manolov and Moeyaert, 2017), yet there is no consensus on the best approach. Approaches using regression coefficients as effect sizes have been devised (Moeyaert et al., 2014; Shadish et al., 2014c). These are able to account for linear or non-linear trends in the data as well as for dependent error structures, however, they are more complicated to implement and interpret, when compared to mean difference based approaches (Heyvaert et al., 2015). Other approaches have been developed and tested using a Bayesian framework (Jones, 2003; Swaminathan et al., 2014; de Vries et al., 2015; Odom et al., 2018), however, implementation is similarly complex. Non-parametric approaches have been proposed such as the Randomization Test Inversion, which exploits the equivalence between a hypothesis test and a Confidence Interval to create an effect size based on the Randomization Test (Michiels et al., 2017), but this is yet to be robustly tested. Tau-U, based on the tradition of examining non-overlap between experimental conditions, combines existing non-parametric tests Mann–Whitney U and the Kendall Rank Correlation coefficient (Parker et al., 2011).

**FIGURE 4:**
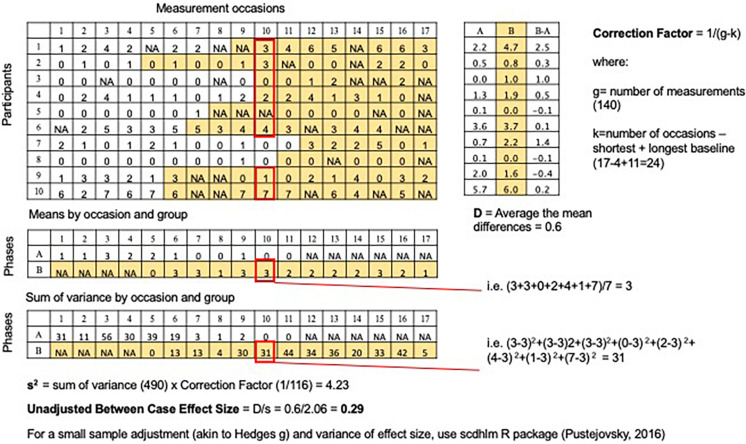
Calculation of unadjusted Between-Case Effect Size. For each measurement occasion, group scores into occasion type (baseline or intervention) and calculate variance; sum all the variances and multiply by a correction factor; take the square root to calculate the denominator (s); numerator is the average mean difference across participants (D); effect size = D/s.

In the current study we focus on the Between-Case Effect Size (BCES) devised by Hedges et al. (2012, 2013) and Pustejovsky et al. (2014), illustrated in Figure 4. The BCES is easy to interpret, has been tested in simulations (Hedges et al., 2012), meta-analyses (Barton et al., 2017), tests of practical applicability (Odom et al., 2018), and comparisons with other approaches (Shadish et al., 2016; Odom et al., 2018). It is accessible to non-statisticians, given the straightforward conceptualization (based on Cohen’s *d*) and the availability of several R packages (Bulté and Onghena, 2009, 2019; Pustejovsky, 2016) and primers (Hedges et al., 2012, 2013; Valentine et al., 2016) to aid calculation.

We applied this approach to evaluate a parent-mediated app-based speech production intervention for minimally verbal autistic preschoolers (*n* = 19). We have recently described the methods, analysis, and challenges to implementing this approach in a population of children that is difficult to recruit and has highly variable patterns of language growth (Saul and Norbury, 2020b). To our knowledge, random assignment and between-case effect size analysis have not previously been applied to a Single Case Experimental Design targeting expressive language growth in minimally verbal autistic children. Single phase was considered the most appropriate format (rather than phase reversal or alternating), since the aim of the intervention is to teach speech sound skills, which once acquired should remain part of the child’s speech sound repertoire. Employing an app-based intervention facilitated remote, repeated sampling of the outcome measure, which is a core component of Single Case Experimental Design. Indeed, the practicality of repeated sampling, and the ability to introduce blinding or independent validation into this process is a key challenge in Single Case Experimental Designs (Smith et al., 2007), which can be addressed using apps in everyday settings.

The overarching goal of the current study is to illustrate how Single Case Experimental Designs with random-assignment can be used to evaluate interventions, particularly for minimally verbal autistic children, by employing the Randomization Test and the Between Case Effect Size. To do this we use real data gathered as part of the BabbleBooster pilot project, with shared data and code (Saul and Norbury, 2020b). We illustrate how in this intervention parents could gather reliable speech attempt data, facilitating remote dense sampling using the app. All objectives and hypotheses relating to the BabbleBooster pilot project were pre-registered^2, 3^.

## Materials and Methods

### Study Design

The study utilized an AB phase design with randomized baseline allocation; the number of weeks of baseline testing (A weeks) and the number of weeks of subsequent intervention (B weeks), were determined randomly for each participant.

Constraints on randomization were as follows:

• each participant had a minimum of three baseline (A) weeks
• each participant had a minimum of six intervention (B) weeks

These constraints were determined due to the limited timeframe available for the intervention (16 weeks), and prioritizing intervention weeks whilst retaining a long enough minimum amount of A weeks for a baseline to be established (Horner et al., 2005). Taking account of these constraints yielded eight possible intervention schedules (Figure 3); a different schedule was randomly assigned to each participant.

### Intervention

The BabbleBooster intervention app was designed to deliver predictable and repetitive speech models via video-modeling and cued articulation (Saul and Norbury, 2020b). The app-play is parent-mediated, so parents are required to watch the stimuli with their children, encourage them to make the sound, and then provide feedback on the accuracy of the production attempt in order to trigger the reward videos. Reward videos were designed with a gradient response, so a ‘good try’ at a sound (an incorrect attempt) will result in a lesser reward than an accurate response. The families were encouraged to make or upload their own reward videos, based on their understanding of the individual child’s interests and reward. Acceptability data and development of the app prototype are discussed in Saul and Norbury (2020b).

### Participants

**FIGURE 5:**
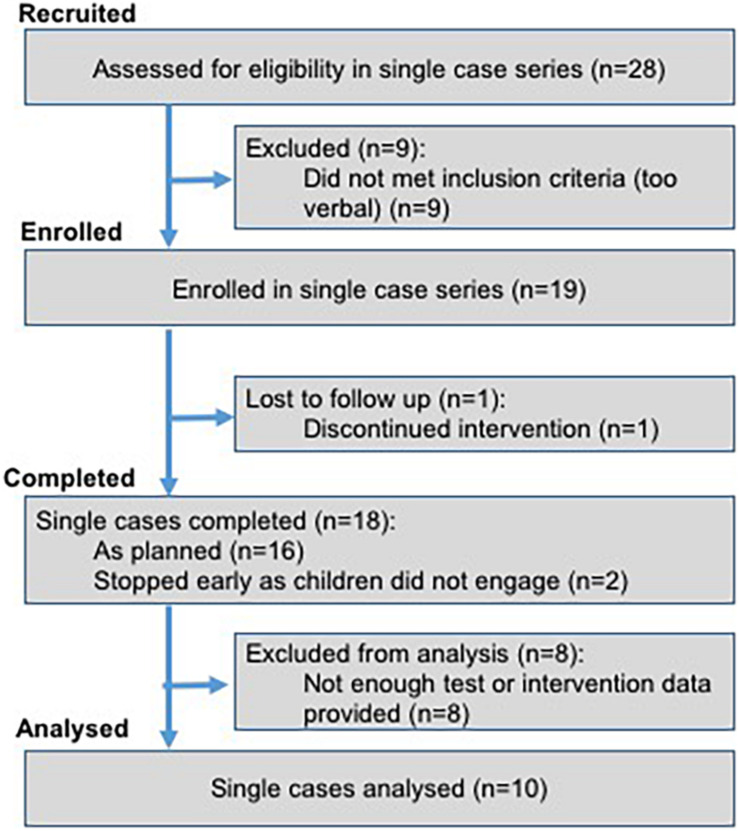
Recruitment flow chart.

Figure 5 describes the process through which participants were selected for the study. Participants were 19 minimally verbal autistic children (three girls, 16 boys) for whom parents reported fewer than 10 sounds or 20 words or produced fewer than five spontaneous words during an initial assessment visit. We gathered quarterly reports on the type and amount of therapy received by each participant. Participants received an average of 0.68 h of Speech and Language Therapy per week (range: 0–2.5 h).

The children were aged 47–74 months at Visit 1 (mean = 60, *SD* = 7) with a confirmed diagnosis of autism. The following exclusions applied at initial screening: epilepsy; known neurological, genetic, visual or hearing problems; English as an Additional Language. Participants were recruited via social media, local charities, independent therapists and a university-run autism participant recruitment agency, and all took part in a larger longitudinal study (Saul and Norbury, 2020a). Ethical approval was obtained from the UCL Research Ethics Committee (Project ID 9733/001) and informed consent was sought from parents on behalf of each participant.

Parents reported 17 participants to be White, one to be Asian and one to be Mixed Race. Eight caregivers had completed high school, eight completed university education and three completed post-graduate studies or equivalent. Eighty-eight percent of parents reported that their child had an Education Health and Care Plan, a legal document that specifies special educational support required for the child, at Visit 1.

### Power

Given the above described constraints (16 weeks of data collection, 8 potential intervention schedules and 19 eligible participants) a sensitivity power analysis was conducted using simulation. One important unknown variable was how correlated dependent variable scores would be within participant, so three scenarios were modeled: low correlation (ICC = 0.25), medium (ICC = 0.50), and high (ICC = 0.75). This suggested adequate power to detect effect sizes of 0.48 and above (high correlation) to 0.84 and above (low correlation), whereas group studies of a comparable size would require larger effect sizes to reach the same power (see Supplementary Appendix B).

### Procedure

Children were seen in their homes for two sessions (Visit 1 and Visit 2), separated by 4 months each (mean = 4.0, *SD* = 0.3). A thank you gift of a small toy or £5 voucher was provided following each visit.

At **Visit 1**, each participant received a new Samsung Galaxy Tab A6 tablet containing the BabbleBooster app^4^, unless parents expressed a preference to use the app on their own Android device (*n* = 3). Parents were given a demonstration of the app by the experimenter, and an information pack explaining how to download and use the app. Secondly, the Probe Phonemes were selected by following the ‘Sound Target Protocol’ (see Supplementary Appendix A) and each parent-child dyad was informed of their randomly allocated intervention start date. Probe Phonemes constituted the outcome variable and comprised nine speech sounds that were elicited each week in the baseline and intervention periods. They also formed the list from which an initial three target phonemes were drawn for the intervention. Probe Phonemes remained the same for each participant and were not manipulated as part of the experiment, rather they were a necessary feature to accommodate the fact that each participant had a unique profile of speech related difficulties.

**Between Visits 1 and 2**, text message reminders were sent to parents to remind them of the weekly probe day, and if necessary, missed probes were rearranged for the following day. Parents also received a reminder text on the intervention start date. Thereafter, parents were asked to engage their child in play with the app for 5–10 min per day, 5 days per week. This resulted in children carrying out the intervention for between 6 and 13 weeks (see Figure 3). For each weekly assessment of the outcome measure, all pertinent information was uploaded to the server [date stamp, phoneme, attempt number, parent rating (either “correct,” “incorrect attempt,” or “no attempt”)] and a video clip of the attempt. Parents pressed one of three buttons to assign a rating to the attempt, in accordance with Table 1.

**TABLE 1:** BabbleBooster parent rating buttons.

Button	Meaning	Example	Consequence
Yes	Child has produced elicited sound accurately	Child is asked to say /b/ and they say /b/	‘Well done’ video
Good Try	Child tried to make a sound but did not make the target sound	child is asked to say /b/ and they say /w/	‘Good try’ video
Try Again	Child does not attempt to make any sound	child is silent/shouts/cries	No video clip

**On Visit 1 and 2**, additional parent-report language measures were obtained to characterize the number of words understood and spoken by the child, as well as direct recording of the number of consonants uttered by the child during a natural language sample (Consonant Inventory).

**Data collected prior to Visit 1:** As the participants were drawn from a previous longitudinal study (Saul and Norbury, 2020a), further background measures, which were gathered between 8 and 12 months prior to the current study, were also available to characterize the sample. Table 2 displays descriptive variables for the intervention group.

**TABLE 2:** Descriptive variables.

Measure	Description	Time	*n*	Mean	*SD*	Min	Max
Age	Age in months	Visit 1	18	61.6	7.5	47.6	74.6
		Visit 2	18	65.7	7.3	52.2	78.3
Receptive language	Oxford CDI words understood (Hamilton et al., 2000) (words)	Visit 1	18	182.2	135.2	5.0	406.0
		Visit 2	18	195.0	141.9	5.0	417.0
Expressive language	Oxford CDI words spoken (Hamilton et al., 2000) (words)	Visit 1	18	4.5	6.4	0.0	19.0
		Visit 2	18	11.6	26.3	0.0	90.0
Consonant inventory	CSBS Scale 11 (Wetherby and Prizant, 2002) (raw score)	Visit 1	18	6.4	3.6	1.0	13.0
		Visit 2	17	5.2	4.4	0.0	16.0
Autism symptom severity	CARS (Schopler et al., 1988) raw score	Time 1	19	42.7	4.9	35.0	52.5
NVIQ	Visual Reception and Fine Motor subtests of Mullen Scales of Early Learning (Mullen, 1995) transformed into Developmental Quotient (developmental age in months/age in months)	Time 2	19	0.36	0.13	0.13	0.56

*CARS, Childhood Autism Rating Scales; CDI, Communicative Development Inventory; CSBS, Communication and Symbolic Behavior Scales; ESCS, Early Social Communication Scales; NVIQ, non-verbal intelligence quotient; SD, standard deviation; Time 1: 12 months prior to Visit 1; Time 2: 8 months prior to Visit 1.*

### Primary Outcome Measure: Elicited Phoneme Weekly Score

Each child received a probe score out of 9 for each of the 16 weeks between Visit 1 and Visit 2. This was used to generate a mean baseline probe score and a mean intervention probe score, as well as the mean difference between these two measures.

#### Missing Data

In the pre-registered analysis, we planned to impute all missing data for the outcome variable following Enders (2010); however, following data collection we made a distinction between participants who did not reliably engage with the testing regime (‘low users’) and those who did (‘high-users,’ who each provided more than 66% of all data points). Results were reported for high-users only, both on the basis of the incomplete dataset and pooled estimates from 40 multiply imputed datasets, created using the Amelia package in R (Honaker et al., 2011). Given that using multiple imputation programs may not be feasible for all clinicians or researchers seeking to use these methods, we provide code with and without imputation in Supplementary Appendix C.

#### Reliability of Parent Ratings

The primary outcome measure is derived from parent ratings of elicited phoneme attempts. To assess reliability of parent scores, 20% of the probes were coded by a qualified Speech and Language Therapist, who was not involved in the study, and was blind to the intervention targets and individual assessment point.

To calculate the reliability of the parent ratings, we derived a list of the filenames of all available video clips downloaded from the BabbleBooster server for the 10 analyzed participants (*n* = 1,120). This number did not correspond with the total number of parent ratings (*n* = 1,248) due to the loss of some videos due to technical problems with the devices used. For coding purposes, data from incomplete weeks were also removed (*n* = 113). Videos were not selected completely at random: the sample needed to include at least 2 complete weeks of data for each user (*n* = 214 videos) since the variable we were comparing across raters was the weekly score. Weeks were chosen at random from the available weeks and comprised at least one A and one B week^5^. For each video clip, the blind coder was told which sound the child was attempting and told to rate it as ‘no attempt,’ ‘incorrect attempt,’ or ‘correct attempt’ in accordance with Table 1, corresponding to a score of 0, 0.5, or 1.

This process generated two to three randomly selected weekly scores for each of the 10 ‘high use’ participants, which were used to compute an intra-class correlation coefficient, using the intra-class correlation ICC() command in the psych R package (Revelle, 2018). An agreement of 0.85 or higher was considered an acceptable level of agreement (Koo and Li, 2016, suggest > 0.75 represents good agreement).

#### Attrition and Adherence

We report adherence to allocated intervention start date for each participant, given its importance to the accuracy of the randomization test. In addition, participants were required to submit > 66% of weekly test data-points to be included in the analysis of primary outcome; proportion of missing data is reported below.

### Analysis Plan

#### Randomization Test

The statistical model used to analyze the significance of a positive change in the primary outcome variable (elicited phoneme test score), was the randomized phase design with resampling as outlined in Rvachew and Matthews (2017). This is a one-tailed analysis, and was calculated in R (R Core Team, 2017) using the script detailed in Supplementary Appendix C. The anonymized dataset is available to download here: https://osf.io/rzuwt/.

*P*-values were pooled across participants, to gauge the consistency of any treatment effects. This was done using the sumz function in the MetaP Package in R (Dewey, 2019), which uses Stouffer’s z-trend procedure to generate a *p*-value that denotes the likelihood of achieving a series of *p*-values merely by chance. We used a *p*-value of less than 0.05 for significance testing for the meta-analysis of *p*-values.

#### Between-Case Effect Size

Between-case Effect Size was calculated for the case series using the ‘scdhlm’ package (Pustejovsky, 2016) and following the guidelines set out in Valentine et al. (2016). Thus performing the command MB_effect_size() generated an adjusted *d* statistic as well as its variance. Sample code is provided in Supplementary Appendix C.

## Results

### Reliability of Parent Ratings of Speech Production Attempts

**FIGURE 6:**
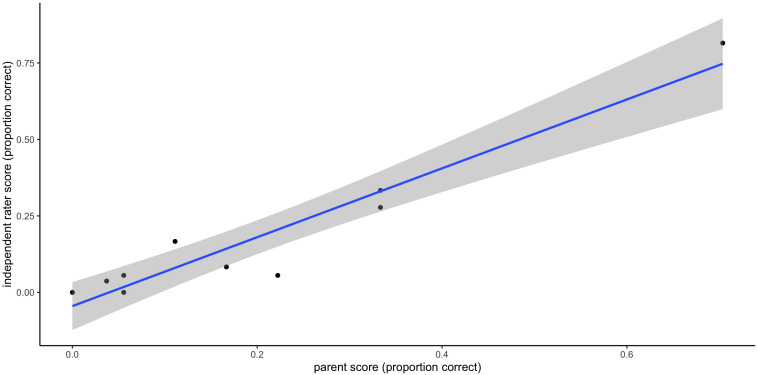
Reliability of parent-rated versus clinician-rated weekly scores.

The intra-class correlation coefficient for speech production ratings by parents compared with those by an independent rater was 0.84 when scores of 0, 0.5, and 1 were considered (0 = no response, 0.5 = incorrect attempt, and 1 = correct). When scores were re-categorized to reflect a binary correct/incorrect split (scores of 1 and 0 respectively, with an incorrect attempt scoring 0 instead of 0.5), this figure rose to 0.95. In light of this, scores of 0 and 1 were used in all subsequent analyses, rather than 0, 0.5, and 1, as originally planned. Individual weekly scores from the reliability analysis are plotted in Figure 6 to demonstrate the level of consistency achieved. The within-participant variability of scores was also of interest, given the importance of stability in the dependent variable to the statistical power suggested in Supplementary Appendix B. One advantage of dense sampling is that it increases power, particularly when each participant’s dependent scores are highly stable. In the current study, each participant supplied at least 12 weeks of probe data; the intra-class correlation coefficient for these scores was 0.75, signifying high consistency in production from week to week.

### Randomization Test

Attrition for the randomization test was 47%, as of the 19 original participants, only 10 were classified as ‘high’ users of the app, insofar as they completed > 66% of test trials. Amongst these high users, the mean number of test trials completed was 82% (*SD* = 11%, range = 69–100%). It was possible to calculate efficacy measures using the data collected from these 10 participants despite the missing data points. Comparison of allocated intervention start date and actual intervention start date revealed a mean delay of 1.4 weeks (*SD* = 1.3, range = 0–3).

**FIGURE 7:**
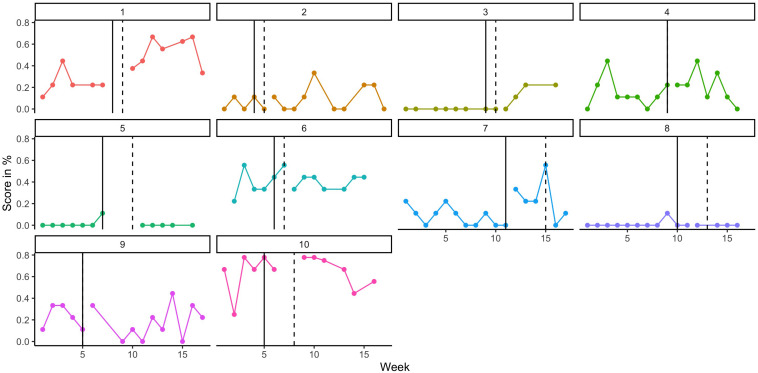
Weekly scores on elicited phoneme test, by participant (as %). The vertical line represents the allocated start week for intervention and the dashed line is the actual start week.

Figure 7 presents the individual weekly probe scores of each participant (score out of 9 expressed as a percentage). These scores were used to compute the mean difference score for each participant and compare it to the distribution of potential outcomes. Intervention was deemed to commence at the actual rather than allocated start date. Table 3 reports each participant’s mean score and standard deviation for A and B weeks, the mean difference between them, and the corresponding rank and *p*-value associated with that mean difference. A non-significant Stouffer’s *Z* statistic was calculated for this range of *p*-values (z = 0.326 *p* = 0.37), indicating that they were not significantly different from *p*-values expected under the null hypothesis. In accordance with the pre-registration, this procedure was also re-run using multiply imputed values, also generating a non-significant result (*z* = –0.115, *p* = 0.91). The same analysis completed using allocated intervention start dates did not result in materially different results (*z* = 0.314, *p* = 0.38).

**TABLE 3:** Comparison of A and B week elicited phoneme scores.

ID	A week mean (*SD*) elicited phonemes (proportion correct)	B week mean (*SD*) elicited phonemes (proportion correct)	Mean difference (B – A weeks)	Rank	*p*-value
1	0.241 (0.109)	0.525 (0.140)	0.284	3	0.375
2	0.044 (0.061)	0.110 (0.122)	0.065	4	0.500
3	0.000 (0.000)	0.139 (0.106)	0.139	2	0.250
4	0.148 (0.136)	0.206 (0.149)	0.058	2	0.250
5	0.016 (0.042)	0.000 (0.000)	–0.016	5	0.625
6	0.407 (0.135)	0.397 (0.059)	–0.011	7	0.875
7	0.148 (0.155)	0.056 (0.079)	–0.093	1	0.125
8	0.009 (0.032)	0.000 (0.000)	–0.009	6	0.750
9	0.222 (0.111)	0.178 (0.159)	–0.044	4	0.500
10	0.642 (0.196)	0.660 (0.137)	0.019	3	0.375

Given the lack of overall treatment effect, further analysis of individual treatment response is unwarranted. In order to demonstrate the feasibility of such analysis we present the individual background characteristics of the ten ‘high user’ participants in Table 4.

**TABLE 4:** Individual characteristics of ‘high users.’

ID	Mean SLT hours/week	Autism Severity (CARS, Time 1)	NIVQ (Time 2)	Age at Visit 1	RCDI at Visit 1	ECDI at Visit 1	Consonant inventory at Visit 1	Age at Visit 2	RCDI at Visit 2	ECDI at Visit 2	Consonant inventory at Visit 2
1	1.00	35	0.38	74.6	68	0	5	78.3	51	0	1
2	1.75	41.5	0.4	61.2	290	0	4	65.1	304	1	7
3	0.66	49	0.49	56.6	5	0	5	61.4	5	0	10
4	0.50	46	0.48	60.3	282	1	7	64.0	277	1	6
5	0.02	48.5	0.28	57.2	38	0	2	60.8	47	0	5
6	0.98	37	0.56	54.4	212	19	4	58.6	224	3	4
7	0.01	43	0.38	69.6	337	0	9	73.3	412	0	7
8	0.38	46.5	0.17	62.6	8	5	12	67.0	11	1	0
9	1.25	46.5	0.13	59.5	55	0	4	63.8	65	0	1
10	0.75	37	0.53	59.8	314	9	6	63.4	327	90	16

*CARS, Childhood Autism Rating Scales; ECDI, Expressive Communicative Development Inventory; NVIQ, non-verbal intelligence quotient; RCDI: Receptive Communicative Development Inventory; SLT, Speech and Language Therapy; Time 1: 12 months prior to Visit 1; Time 2: 8 months prior to Visit 1.*

### Between Case Effect Size

The Between-Case Effect Size for the above data (*n* = 10), adjusted for small sample size, is 0.267 with a variance of 0.011 (see Supplementary Appendix C for sample code). This small effect size is consistent with the non-significant main finding. Studies have found that single case series often generate larger effects than those expected for group designs, and these effects vary widely depending on the technique used (Parker et al., 2005). In this context, the small effect size does not appear to be clinically meaningful.

## Discussion

The current study sought to describe and illustrate two powerful techniques for statistical analysis of Single Case Experimental Designs, which can be employed where the gold standard RCT may be difficult to implement. We used data from a brief intervention, which aimed to increase speech production skills in minimally verbal autistic children. The randomization test was used to compare the degree of improvement observed during the intervention period to the degree of change possible under the null hypothesis. This test indicated that results were consistent with the null hypothesis (no effect of intervention), with a corresponding small between-case effect size.

Although the intervention did not work as hoped, clearly the method has been useful and has provided insights into reasons why the intervention was not successful. An important factor that has become clear since the study was designed is the sheer volume of input and practice required to effect even a tiny change in expressive language in this population (e.g., Esch et al., 2009; Chenausky et al., 2016). The current study was limited by a 16-week timeframe that also included a baseline of a variable length, thus limiting the number of weeks of intervention. Future studies will require a longer time period to determine optimal treatment intensity and duration, and randomized case series with varying intervention periods are an ideal way to manipulate dosage and inform future larger scale trials.

A second key consideration for future replications is attrition. Our power analyses assumed a starting sample size (*n* = 18), however, only 10 children provided enough data for analysis, resulting in much lower power to detect statistically significant effects. Based on parent feedback, we expect that some attrition was related to frustration with technical difficulties. Due to the design of this study, those not engaging with the app could not be replaced. A major strength of this design is that it does not require baselines to be sequential; thus in future studies replacement could be used to manage attrition. Important considerations for future research also include specifying in pre-registration protocols how best to deal with missing data and adherence to intervention start date, in order to reduce bias in analysis.

The current study has laid useful groundwork for future replications in that we have demonstrated that an app can be used to elicit and record speech production attempts, and parents were able to accurately rate those attempts online following brief training. This means that one can have confidence in parent ratings, and they can be used to evaluate interventions, enhancing the scalability of this, and other apps. We also have an indication of how stable such attempts are in children who met criteria for minimal language, and what percentage of recruited families were able to meet the demands of the testing regime and comply with the intervention schedule. We have been able to illustrate individual differences in treatment response (Figure 7), and had we observed a meaningful treatment response we could have related this to individual child factors (Table 4). What we have demonstrated is that the chosen study design (multiple baseline with random assignment) and statistical approaches (Randomization Test and BCES) are feasible and straightforward to implement with real-world data, as generated by this sample of 10 participants. Based on our initial sample size and power calculations in Supplementary Appendix B, these methods are also more statistically robust than a comparable group study would be.

Randomized case series have a number of additional advantages. Firstly, they provide a much needed boost to power when compared with group designs, meaning that informative results can be obtained with fewer participants. This is critical for neurodevelopmental conditions that make obtaining a large and homogenous cohort challenging. Secondly, these designs are able to elucidate individual differences in treatment response, in a way that larger group studies cannot. Thirdly, case series are inherently a more feasible, low-cost, flexible endeavor, meaning they can be combined with clinical work and executed in a piecemeal fashion over a longer period. Finally, thanks to meta-analytic advances we can combine results from multiple case series in order to draw more robust conclusions about intervention efficacy.

## Conclusion

The goal of this paper was to outline how to implement Single Case Experimental Design, by using random-assignment and the randomization test, as well as a between-case effect size to measure functional relationships between the introduction of an intervention and the outcome variable. The current study demonstrates that this is a robust method for rare, heterogeneous groups. While the BabbleBooster intervention did not lead to meaningful change in spoken language skills on this occasion, our goal is that this study will serve as a template for future studies that seek to answer a range of different therapeutic questions. Additionally, broader adoption of these methods will facilitate meta-analyses, allowing the field to progress in understanding components of effective treatments for improving language in autism and other neurodevelopmental conditions.

The key take away points for any future students, researchers or clinicians seeking to adopt these methods are as follows: Firstly, plan for how many participants will be able to include, and how many times the dependent variable will be measured. These will likely be a function of funding or time constraints, and both have important implications for power. Within the overall study period, consider the minimum and maximum acceptable baseline periods. The maximum baseline will depend on participants’ tolerance of repeated probes (boredom, irritability, practice effects) and the minimum intervention period is that which is expected to yield a meaningful intervention effect. A further planning issue is the number of probe items, how these are allocated and whether they include control items or randomization (see Howard, Best, and Nickels for further discussion of these issues). When it comes to selecting outcome measures, it is important to consider their reliability. In this study, we established parent/clinician reliability for coding speech attempts, which enhanced the scalability of the project by eliminating the need for the researcher to administer all test probes. Future studies will need to check the reliability of other combinations of delivery agents and language measures prior to data collection. Decide in advance how to handle missing data (how much missing data would exclude that participant’s contribution?) or variations in adherence to intervention schedules. Finally, stability of the dependent variable is an important factor. If this is unknown and piloting is not feasible, power sensitivity analyses should take into account the impact of different correlations of the dependent variable at multiple testing points.

Ultimately, we would encourage clinicians and researchers to plan a study that is feasible for them, but to be realistic that they may not achieve adequate power in one “shot.” However, if the studies are executed using the recommended techniques, alongside principles of reproducible open science, they are still valuable because they may be replicated at a later date by the same or different researchers. Lakens (2020) makes these points and adds that there is an ethical component to ensuring that the data we can feasibly collect is done in a way that leads to informative conclusions, either immediately or as part of subsequent meta-analysis. A huge challenge for the field is that RCTs are not always possible, yet single case studies alone are uninformative. However, by using the procedures outlined above we may be able to combine smaller studies through collaboration with other labs or clinics to yield informative conclusions, about intervention effectiveness and individual differences in treatment response.

## Data Availability Statement

The datasets presented in this study can be found in online repositories. The names of the repository/repositories and accession number(s) can be found below: https://osf.io/rzuwt/.

## Ethics Statement

The studies involving human participants were reviewed and approved by UCL Research Ethics Committee. Written informed consent to participate in this study was provided by the participants’ legal guardian/next of kin.

## Author Contributions

JS had primary responsibility for study design, data collection, data analysis, and preparation of the manuscript. CN contributed to study design, oversaw data collection and data analysis, and provided detailed comments on drafts of the manuscript. Both authors contributed to the article and approved the submitted version.

## Conflict of Interest

The authors declare that the research was conducted in the absence of any commercial or financial relationships that could be construed as a potential conflict of interest.

## Footnotes

1 In this article, we use identity-first language (e.g., “autistic individual”) rather than person-first language (e.g., “individual with autism”), as this has been highlighted as the preference of the majority autistic individuals and their families (Kenny et al., 2016).

2 https://osf.io/9gvbs

3 In light of non-significant main findings, the final section of pre-registered analyses was not carried out, as these sought to identify potential moderators of success.

4 One participant received a comparable second hand Nexus 7 tablet.

5 Not possible for one participant due to technical problems with uploading in initial weeks.
