# Supplementary material for: A Randomized Case Series Approach to Testing Efficacy of Interventions for Minimally Verbal Autistic Children
Source: Front Psychol. 2021 May 24;12:621920. doi: 10.3389/fpsyg.2021.621920 (PMC8182798; doi:10.3389/fpsyg.2021.621920)
Supplement: Supplementary file 1 [file Table_1.DOCX]

Appendix A: Sound Target Protocol

This decision tree is designed to generate a list of 9 selected sounds for each child from which target sounds will be drawn. The first three sounds on the list will be the first three targets and from then on, any replacements will go down the list in order. It is not expected that any child will already be able to produce more than 4 of the sounds mentioned below, therefore each child will have nine sounds on their list.

Decision tree: Stop when 9 unique sounds have been selected.
