# Supplementary material for: A Randomized Case Series Approach to Testing Efficacy of Interventions for Minimally Verbal Autistic Children
Source: Front Psychol. 2021 May 24;12:621920. doi: 10.3389/fpsyg.2021.621920 (PMC8182798; doi:10.3389/fpsyg.2021.621920)
Supplement: Supplementary file 2 [file Table_2.DOCX]

Appendix B: Simulation

Hypothetical datasets were simulated 100 times each in R (using code below), for a range of effect sizes (.5, .75, 1 and 1.25) with the following assumptions:

- 18 participants
- alpha of .05
- 16-week experimental period with a minimum of 3 baseline (A) and 6 intervention (B) weeks, resulting in 8 permutations of A and B weeks

Intra-class correlation between scores of each participant was either assumed to be .25 (low correlation) .50 (medium correlation) or .75 (high correlation), to model different assumptions about how similar within participant scores are to each other, as greater correlation leads to greater power.

As well as modelling the power of a randomization test to detect a real treatment effect under these parameters, we calculated a comparable power metric for the same dataset, assuming a group design. To do this we randomly assigned half the participants to a control group and half to an intervention group. Mean initial week baseline score is subtracted from mean final week baseline score in the control group (no mean treatment effect expected) and mean initial week baseline score is subtracted from mean final week intervention score in the intervention group. The two group mean differences are then compared using an independent t-test.

Simulation results are presented in Figure I.


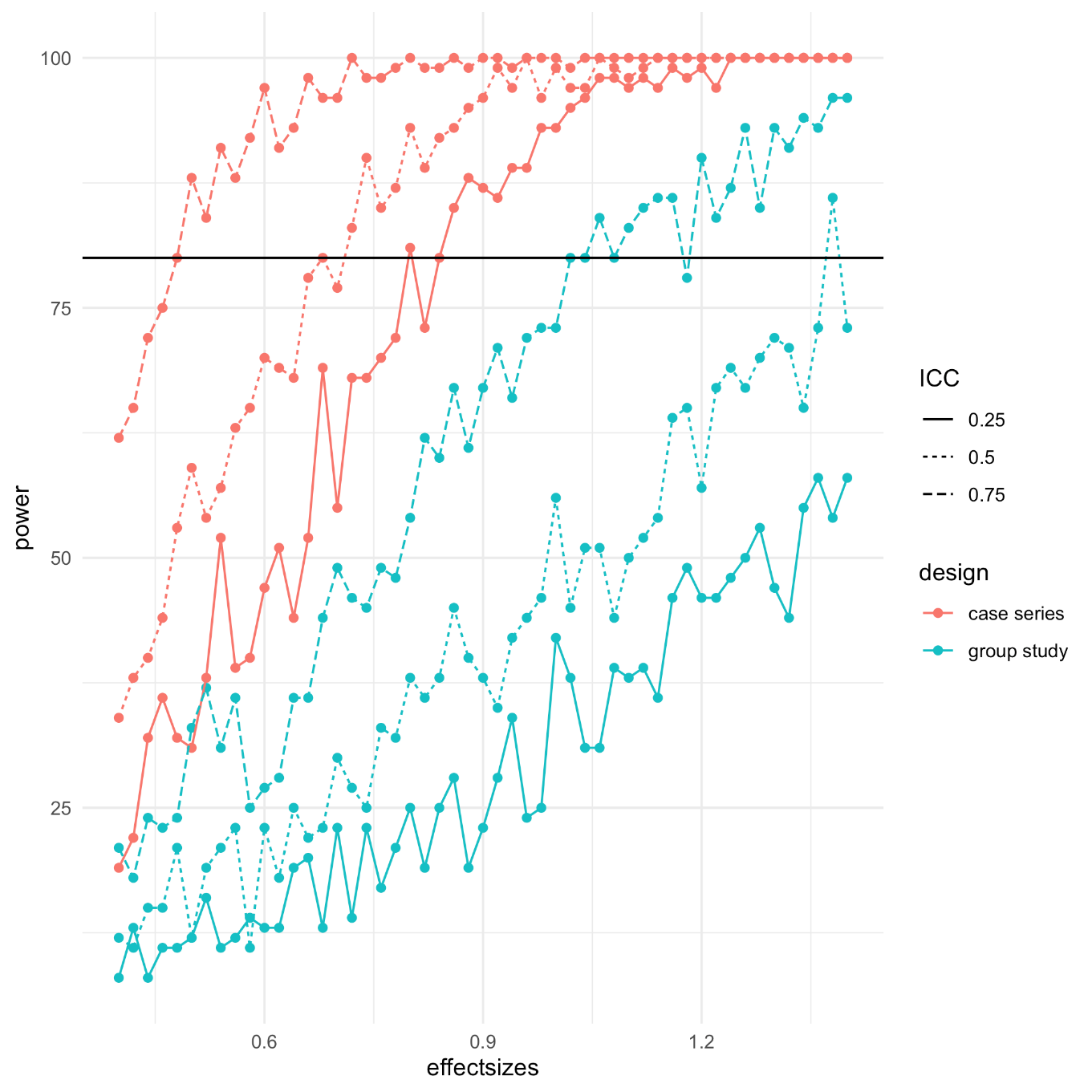


**Figure I**

Power to detect treatment effects of different sizes, assuming n=18, 100 simulations, 8 permutations of A and B weeks, alpha=.05. Blue lines represent power in group design, red lines represent randomized case series design. Solid line assumes ICC=.25 (low correlation), dotted line assumes ICC=.5 (medium correlation) and dashed line assumes ICC=.75 (high correlation)

From the above graph, we conclude that both analysis methods (randomisation test and independent t-test) are sensitive to the correlation or stability of the dependent variable. Furthermore, the randomized case series design has adequate power to detect a real effect size of .68 or more, whereas for the group design an effect size of 1.38 is required to achieve adequate power, assuming medium correlation.

We also illustrate an example dataset (from one simulation, effect size = 0.75) for each of the high and low correlation scenarios in Figures II to V.


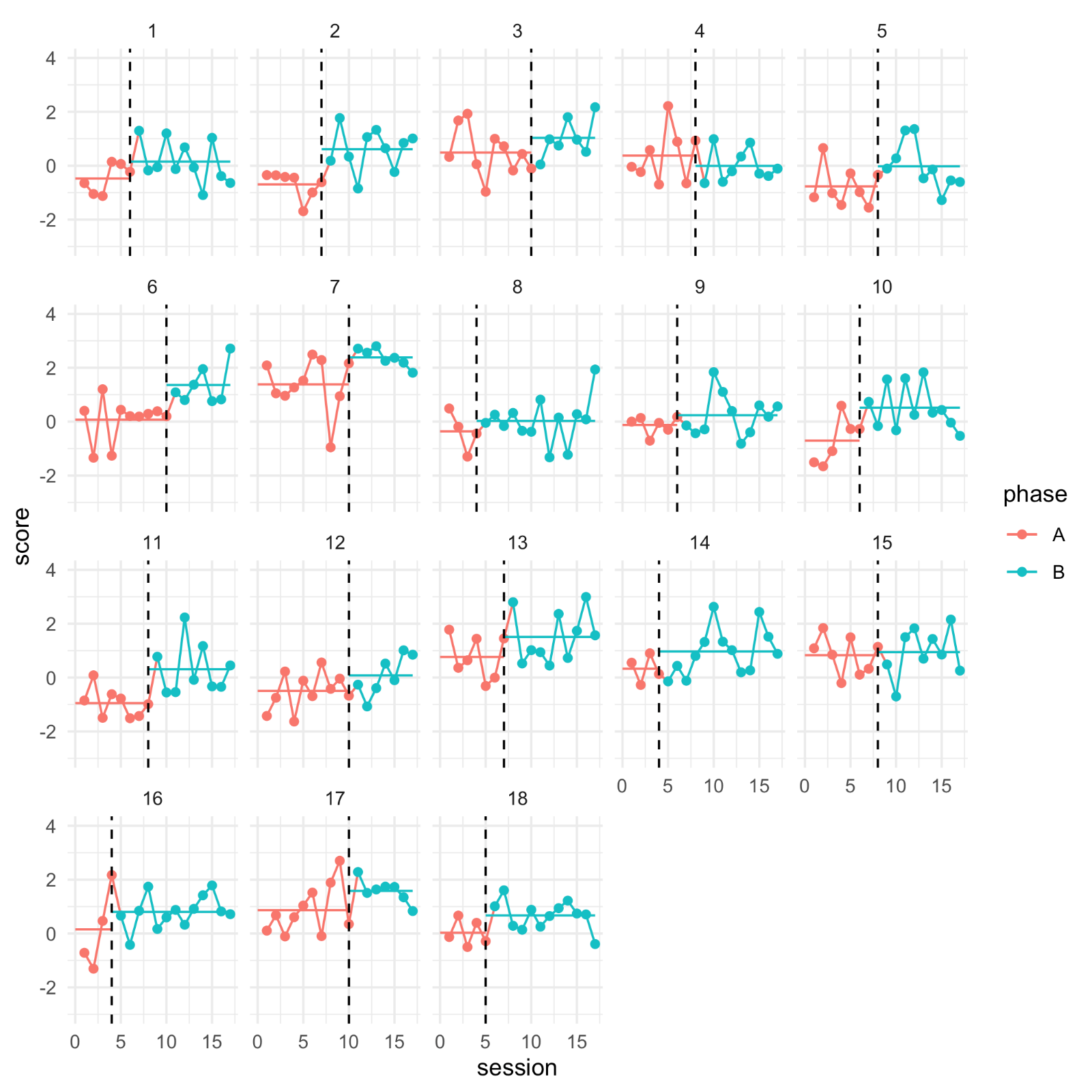


**Figure II. Example of low correlation simulation scores over time (ICC=.25)**

Z scores over time in weeks, each panel represents one participant, vertical line indicates intervention start, horizontal lines represent mean A week and B week scores


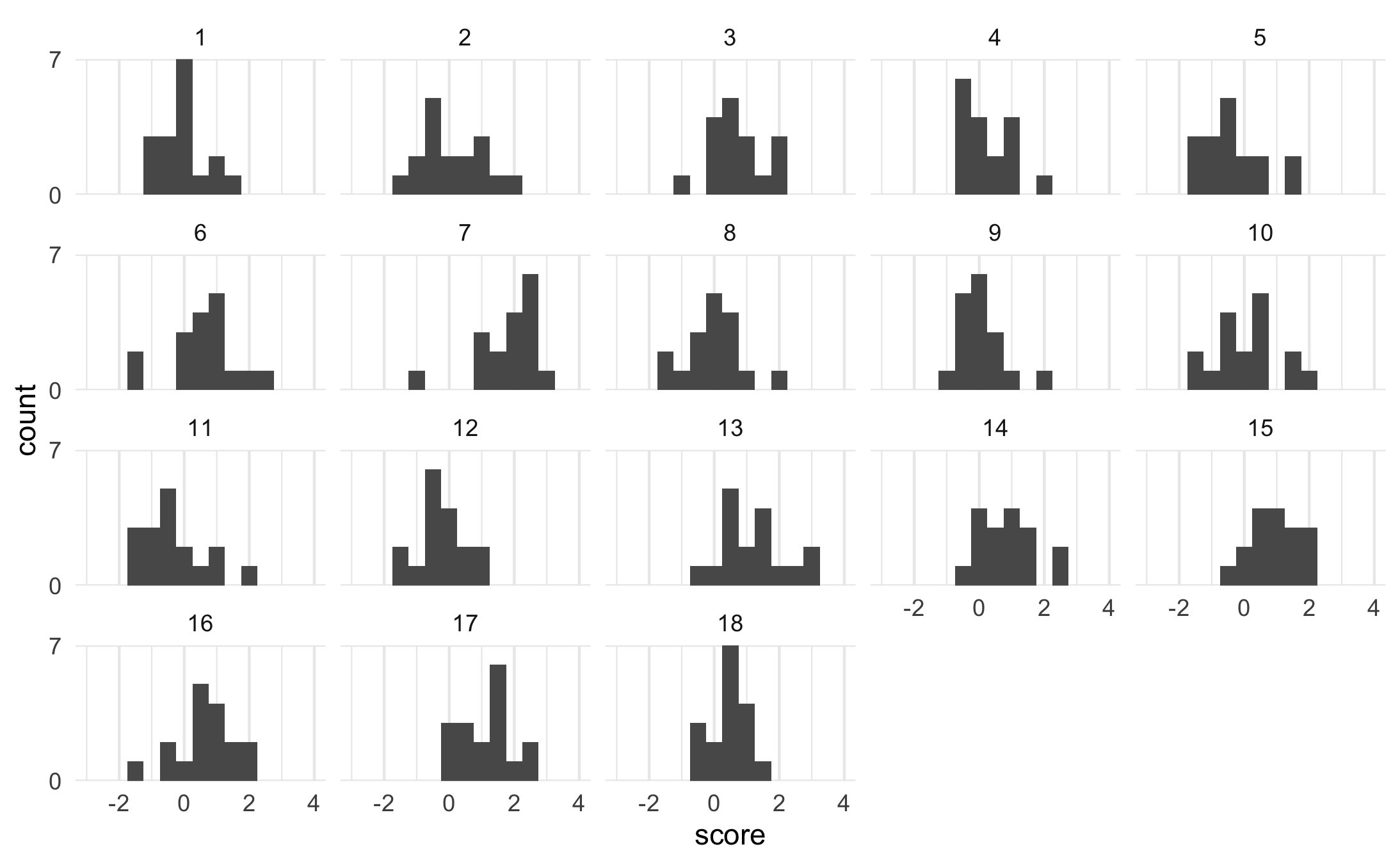


**Figure III. Example of low correlation simulation scores histogram (ICC=.25)**

Z scores distribution, each panel represents one participant


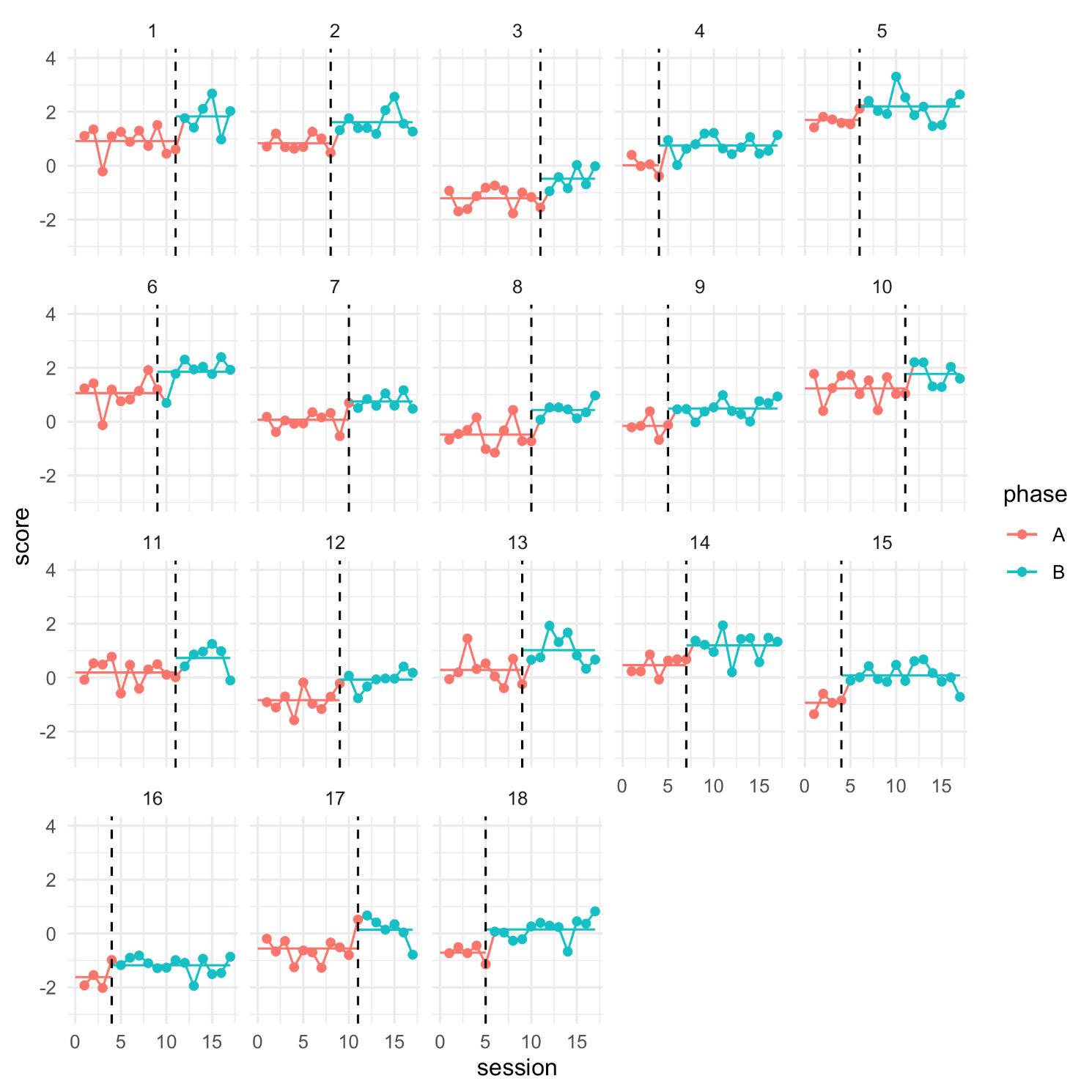


**Figure IV. Example of high correlation simulation scores over time (ICC=.75)**

Z scores over time in weeks, each panel represents one participant, vertical line indicates intervention start, horizontal lines represent mean A week and B week scores


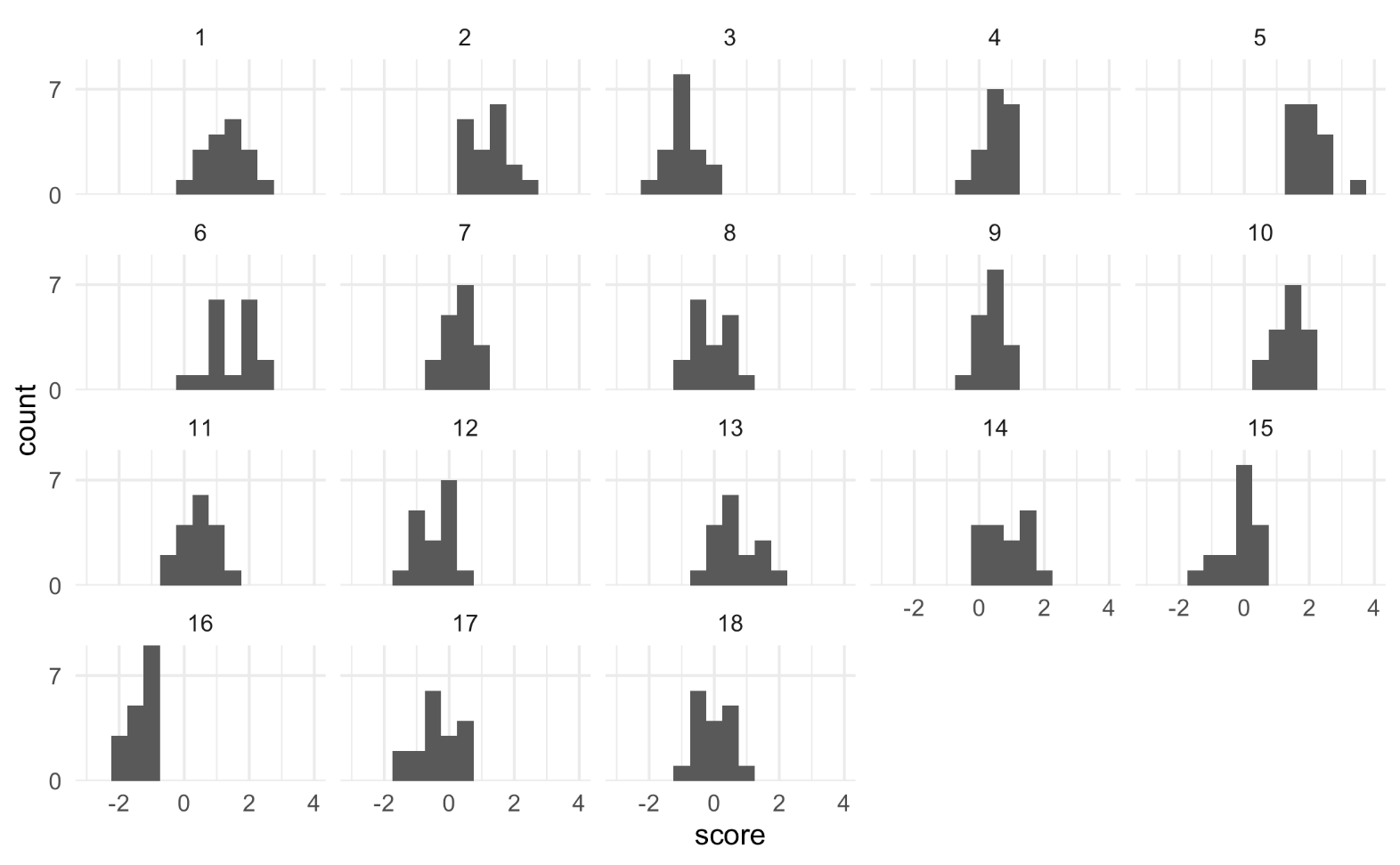


**Figure V. Example of high correlation simulation scores histogram (ICC=.75)**

Z scores distribution, each panel represents one participant

**Simulation Code**

#----------------------------------------

# 1. Install and load all libraries needed

#----------------------------------------

install.packages('tidyverse') # needed for data wrangling

library(tidyverse)

require(devtools) # needed to install dependency of metap that is no longer available:

install_version("multtest", version = "2.8.0", repos = "http://cran.us.r-project.org")

library(multtest)

install.packages('metap') #for Stouffer's z calculation

library(metap)

install.packages('scdhlm') #for between-case effect size calculation

library(scdhlm)

install.packages("multilevel") #needed for icc.sim()

library(multilevel)

#----------------------------------------

# 2. Record function

#----------------------------------------

get_power <- function(myM2, myICC) {

Rand <- tibble(id=1:myN, rand=sample(1:(PermissibleNo), myN, replace=TRUE),) # assigns each participant a random schedule

dfScore1 <- sim.icc(TotalSessions, 1, myICC, nitems=myN,item.cor=myCor) #multilevel package simulates scores with given icc

dfScore1$GRP <- NULL # don't need this column

dfScore1 <- as.data.frame(t(dfScore1)) %>% scale() %>% as.data.frame() # creates the df for baseline score

dfScore1 <- dfScore1 %>% mutate(id=1:myN) %>% gather(key='session', value='score_A', 1:TotalSessions) %>% mutate(score_B=(score_A+myM2)) %>% mutate(session=substr(session,2,4) %>% as.numeric()) # adds effect size for intervention score

dfScore1 <- dfScore1 %>% inner_join(Rand, by='id') # combine actual scores with actual schedule allocation

dfScore1 <- dfScore1 %>% inner_join(Permissible, by='session') # combines this with all possible schedules

dfScore1$phase <- NULL # create a phase column for actual allocation of phases

for (i in 1:(myN*TotalSessions) ) {

dfScore1$phase[i] <- dfScore1[i,(dfScore1$rand[i]+5)]

}

data <- dfScore1 %>% mutate(score=if_else(phase=='A',score_A, score_B)) # allocates baseline or intervention score by phase

MD_actual <- data %>% group_by(id, phase) %>%

summarise(score=mean(score, na.rm=TRUE)) %>%

spread(key=phase, value=score) %>%

mutate(mean_diff=B-A) #calculate actual mean diff

MD_all <- data %>% gather(key='sched', value='phase', 6:(5+PermissibleNo))

MD_all <- MD_all %>% group_by(id, sched, phase) %>%

summarise(score=mean(score, na.rm=TRUE)) %>%

spread(key=phase, value=score) %>%

mutate(diff2=B-A) #calc all other mean diffs

# compare actual MD to all MD for each participant

MD_all <- inner_join(MD_all, MD_actual, by='id') #join data together

MD_all <- MD_all %>% mutate(n=(mean_diff)>=(diff2)) #mark true or false if actual MD equal to or greater than the hypothetical one

pvals <- MD_all %>% ungroup() %>% group_by(id) %>% summarise(pval=(PermissibleNo+1-sum(n))/PermissibleNo) #turn this into a pvalue

#pool pvalues

p_case_series <- sumz(pvals$pval)$p %>% as.numeric()#Stouffer's Z from the metap package

# work out group equivalent p-values, assuming first half of n is control (take both score_A) and second is intervention take score_A and then score_B

control <- data %>% filter(id %in% 1:(myN/2)) %>%

filter(session %in% c(1, TotalSessions)) %>%

dplyr::select(id, session, score_A) %>%

spread(session, score_A)

colnames(control) <- c('id', 'pre','post')

control <- control %>% mutate(meandiff=post-pre)

intervention <- data %>% filter(id %in% (myN/2+1):TotalSessions) %>%

filter(session %in% c(1, TotalSessions)) %>%

dplyr::select(id, session, score) %>%

spread(session, score)

colnames(intervention) <- c('id', 'pre','post')

intervention <- intervention %>% mutate(meandiff=post-pre)

ttest <- t.test(control %>% pull(meandiff), intervention %>% pull(meandiff))

p_group <- ttest$p.value

#output p-value for randomised case series and group equivalent

output <- c(p_case_series, p_group)

return(output)

}

#-----------------------------------------------------------------------------------

# 3. Input minimum lengths for A and B phase and how many measurement sessions in total

#-----------------------------------------------------------------------------------

PhaseAMin = 4 # baseline for first 4 weeks

PhaseBMin= 6 # intervention for last 6 weeks

TotalSessions = 17 # 17 weeks in total

#-----------------------------------------------------------------------------------

# 4. Input simulation parameters

#-----------------------------------------------------------------------------------

myN <- 18 # number of cases in the series, i.e. your planned number of participants, must be even number

mySim <- 100 # number of simulations you want to run

myAlpha <- 0.05 # the alpha level for power calc

myM2 <- 0.75 # intervention mean score (equates to effect size given that baseline has mean 0 and SD of 1)

myICC <- 0.75 # correlation within participants

myCor <- 0 # correlation between participants

#-----------------------------------------------------------------------------------

# 5. Calculate the Permissible schedules and combine them with the actual data

#-----------------------------------------------------------------------------------

PermissibleNo <- TotalSessions-PhaseAMin - PhaseBMin + 1 # number of Permissible schedules

Permissible <- tibble(n=1:TotalSessions) #create a table of all scheduling options

for (i in 1:PermissibleNo)

{

Permissible[i] <-c(rep('A', PhaseAMin),rep('A',(i-1)), rep('B',PermissibleNo-(i-1)), rep('B',PhaseBMin-1))

}

colnames(Permissible) <- paste('sched', 1:PermissibleNo,sep="") #rename the schedules

Permissible <- Permissible %>% mutate(session=1:(TotalSessions)) #add session numbers

#-----------------------------------------------------------------------------------

# 6. Calculate Power

#-----------------------------------------------------------------------------------

results <- replicate(mySim,get_power(myM2, myICC)) %>% t() %>% as.tibble()

colnames(results) <- c('p1', 'p2')

p_case <- results %>% mutate(sig=(p1<=myAlpha)) %>% summarise(power=mean(sig)) *100

p_group <- results %>% mutate(sig=(p2<=myAlpha)) %>% summarise(power=mean(sig)) *100

p_case # displays the randomised case design power

p_group # displays the group design power
