# Supplementary material for: A Randomized Case Series Approach to Testing Efficacy of Interventions for Minimally Verbal Autistic Children
Source: Front Psychol. 2021 May 24;12:621920. doi: 10.3389/fpsyg.2021.621920 (PMC8182798; doi:10.3389/fpsyg.2021.621920)
Supplement: Supplementary file 3 [file Table_3.docx]

Appendix C: Code to Calculate Randomisation Test

#--------------------------------------

# 1. Install and load all libaries needed

#--------------------------------------

install.packages('tidyverse') # needed for data wrangling

library(tidyverse)

require(devtools) # needed to install dependency of metap that is no longer available:

install_version("multtest", version = "2.8.0", repos = "http://cran.us.r-project.org")

library(multtest)

install.packages('metap') #for Stouffer's z calculation

library(metap)

install.packages('scdhlm') #for between-case effect size calculation

library(scdhlm)

#--------------------------------------------------------------------------

# 2. Read in the data from the study (find this file on OSF page or use your own data)

#--------------------------------------------------------------------------

data_orig <- read.csv('OSF_datafile.csv') # this needs to be organised with 4 columns: id, phase, score and session number

#--------------------------------------------------------------------------

# 3. Input minimum lengths for A and B phase and how many measurement sessions in total

#--------------------------------------------------------------------------

PhaseAMin = 4 # baseline for first 4 weeks

PhaseBMin= 6 # intervention for last 6 weeks

TotalSessions = 17 # 17 weeks in total

#--------------------------------------------------------------------------

# 4. Calculate the permissible schedules and combine them with the actual data

#--------------------------------------------------------------------------

PermissibleNo <- TotalSessions-PhaseAMin - PhaseBMin + 1 # number of permissible schedules

#create a table of all scheduling options

Permissible <- tibble(n=1:TotalSessions)

for (i in 1:PermissibleNo) {

Permissible[i] <-c(rep('A', PhaseAMin),rep('A',(i-1)), rep('B',PermissibleNo-(i-1)), rep('B',PhaseBMin-1))

}

colnames(Permissible) <- paste('sched', 1:PermissibleNo,sep="") #rename the schedules

Permissible <- Permissible %>% mutate(session=1:(TotalSessions)) #add session numbers

data <- inner_join(data_orig, Permissible, by='session') #join with original data

#--------------------------------------------------------------------------

# 5. calculate actual mean difference

#--------------------------------------------------------------------------

MD_actual <- data %>% group_by(id, phase) %>%

summarise(score=mean(score, na.rm=TRUE)) %>%

spread(key=phase, value=score) %>%

mutate(mean_diff=B-A)

#--------------------------------------------------------------------------

# 6. calculate all other mean differences

#--------------------------------------------------------------------------

MD_all <- data %>% gather(key='sched', value='phase', 5:(4+PermissibleNo))

MD_all <- MD_all %>% group_by(id, sched, phase) %>%

summarise(score=mean(score, na.rm=TRUE)) %>%

spread(key=phase, value=score) %>%

mutate(diff2=B-A)

#--------------------------------------------------------------------------

# 7. compare actual MD to all MD for each participant

#--------------------------------------------------------------------------

MD_all <- inner_join(MD_all, MD_actual, by='id') #join data together

MD_all <- MD_all %>% mutate(n=(mean_diff)>=(diff2)) #mark true or false if actual MD equal to or greater than the hypothetical one

pvals <- MD_all %>% ungroup() %>% group_by(id) %>% summarise(pval=(PermissibleNo+1-sum(n))/PermissibleNo) #turn this into a pvalue

#--------------------------------------------------------------------------

# 8. Pool pvalues

#--------------------------------------------------------------------------

sumz(pvals$pval) #Stouffer's Z from the metap package

#--------------------------------------------------------------------------

# 9. Calculate effect size

#--------------------------------------------------------------------------

data_orig <- data_orig %>% na.omit() #no missing values are allowed

ES <-effect_size_MB(data_orig$score, data_orig$phase, data_orig$id, data_orig$session) # calculates between-case effect size

ES$delta_hat # d statistic (Adjusted for small samples)

ES$V_delta_hat # variance of d

# Extra code to include if multiple imputation is used

```{r}

install.packages("Amelia")

library(Amelia)

#1. create bounds for Amelia to use when guessing the score values (range from 0% to 100% treat as continuous. 1st argument is column number, then lower then upper limit for values

bds <- matrix(c(4, 0, 1), nrow = 1, ncol = 3)

#2. Run the multiple imputation, which creates m imputation sets limited by bounds bds and subject to maximum of 1000 attempts.

#First the variable phase needs to be converted to a numeric value

data_mult <- data_orig %>% mutate(phase=dplyr::recode(phase, 'A'=0,'B'=1))

a.out1 <- amelia(data_mult, m=40, ts = "session", cs = "id", bounds = bds,

max.resample = 1000)

#3. Create a loop for k imputations, here k=40 (m=40 above), each imputed dataset is used to generate a z and p value, which is stored in Results.

myImps <- 40

#create the df for results to be stored from each imputation

Results <-data.frame(matrix(data=NA,nrow=myImps,ncol=3))

colnames(Results) <-c("m","z","p") #z and p are empty before we run the loop

Results$m <-1:myImps # imputation number

#start the loop

for (k in 1:myImps)

{

#pull one of the imputations

data_imp <- a.out1$imputations[[k]]

#--------------------------------------------------------------------------

# Create the Permissable schedules df (as per above)

#--------------------------------------------------------------------------

PhaseAMin = 4 # baseline for first 4 weeks

PhaseBMin= 6 # intervention for last 6 weeks

TotalSessions = 17 # 17 weeks in total

PermissibleNo <- TotalSessions-PhaseAMin - PhaseBMin + 1

Permissible <- tibble(n=1:TotalSessions)

for (i in 1:PermissibleNo) {

Permissible[i] <-c(rep('A', PhaseAMin),rep('A',(i-1)), rep('B',PermissibleNo-(i-1)), rep('B',PhaseBMin-1))

}

colnames(Permissible) <- paste('sched', 1:PermissibleNo,sep="") #rename the schedules

Permissible <- Permissible %>% mutate(session=1:(TotalSessions)) #add session numbers

#convert phase variable back to A and B factors in order to run the imputation

data <- data_imp %>% mutate(phase=dplyr::recode(phase, '0' = 'A', '1' = 'B'), phase=as.factor(phase)) %>% #switch back to A/B phases

inner_join(Permissible, by='session') #join with Permissable schedules

#--------------------------------------------------------------------------

# calculate actual mean difference (as per above)

#--------------------------------------------------------------------------

MD_actual <- data %>% group_by(id, phase) %>%

summarise(score=mean(score, na.rm=TRUE)) %>%

spread(key=phase, value=score) %>%

mutate(mean_diff=B-A)

#--------------------------------------------------------------------------

# calculate all other mean differences (as per above)

#--------------------------------------------------------------------------

MD_all <- data %>% gather(key='sched', value='phase', 5:(4+PermissibleNo))

MD_all <- MD_all %>% group_by(id, sched, phase) %>%

summarise(score=mean(score, na.rm=TRUE)) %>%

spread(key=phase, value=score) %>%

mutate(diff2=B-A)

#--------------------------------------------------------------------------

# compare actual MD to all MD for each participant (as per above)

#--------------------------------------------------------------------------

MD_all <- inner_join(MD_all, MD_actual, by='id') #join data together

MD_all <- MD_all %>% mutate(n=(mean_diff)>=(diff2)) #mark true or false if actual MD equal to or greater than the hypothetical one

pvals <- MD_all %>% ungroup() %>% group_by(id) %>% summarise(pval=(PermissibleNo+1-sum(n))/PermissibleNo) #turn this into a pvalue

#--------------------------------------------------------------------------

# Pool pvalues (as per above)

#--------------------------------------------------------------------------

mySumz <- sumz(pvals$pval) #Stouffer's Z from the metap package

#save the resulting Z and p values for this imputation and move on to the next one

Results$p[k] <- as.numeric(mySumz$p)

Results$z[k] <- mySumz$z

}

# 4. Once all imputations have been run, pool results using Rubin's rules

z <- mean(Results$z) #average z score across k imputations #

pvalue <- 2*pnorm(abs(z), lower.tail = F) #p value assuming 2 tailed distribution

**Alternative Code for Multiple Imputation**

install.packages("Amelia")

library(Amelia)

#1. create bounds for Amelia to use when guessing the score values (range from 0% to 100% treat as continuous. 1st argument is column number, then lower then upper limit for values

bds <- matrix(c(4, 0, 1), nrow = 1, ncol = 3)

#2. Run the multiple imputation, which creates m imputation sets limited by bounds bds and subject to maximum of 1000 attempts.

#First the variable phase needs to be converted to a numeric value

data_mult <- data_orig %>% mutate(phase=dplyr::recode(phase, 'A'=0,'B'=1))

a.out1 <- amelia(data_mult, m=40, ts = "session", cs = "id", bounds = bds,

max.resample = 1000)

#3. Create a loop for k imputations, here k=40 (m=40 above), each imputed dataset is used to generate a z and p value, which is stored in Results.

myImps <- 40

#create the df for results to be stored from each imputation

Results <-data.frame(matrix(data=NA,nrow=myImps,ncol=5))

colnames(Results) <-c("m","z","p", "d", "d_V") #z, p, d and d_V are empty before we run the loop

Results$m <-1:myImps # imputation number

#start the loop

for (k in 1:myImps)

{

#pull one of the imputations

data_imp <- a.out1$imputations[[k]]

#--------------------------------------------------------------------------

# Create the Permissible schedules df (as per above)

#--------------------------------------------------------------------------

PhaseAMin = 4 # baseline for first 4 weeks

PhaseBMin= 6 # intervention for last 6 weeks

TotalSessions = 17 # 17 weeks in total

PermissibleNo <- TotalSessions-PhaseAMin - PhaseBMin + 1

Permissible <- tibble(n=1:TotalSessions)

for (i in 1:PermissibleNo) {

Permissible[i] <-c(rep('A', PhaseAMin),rep('A',(i-1)), rep('B',PermissibleNo-(i-1)), rep('B',PhaseBMin-1))

}

colnames(Permissible) <- paste('sched', 1:PermissibleNo,sep="") #rename the schedules

Permissible <- Permissible %>% mutate(session=1:(TotalSessions)) #add session numbers

#convert phase variable back to A and B factors in order to run the imputation

data <- data_imp %>% mutate(phase=dplyr::recode(phase, '0' = 'A', '1' = 'B'), phase=as.factor(phase)) %>% #switch back to A/B phases

inner_join(Permissible, by='session') #join with Permissable schedules

#--------------------------------------------------------------------------

# calculate actual mean difference (as per above)

#--------------------------------------------------------------------------

MD_actual <- data %>% group_by(id, phase) %>%

summarise(score=mean(score, na.rm=TRUE)) %>%

spread(key=phase, value=score) %>%

mutate(mean_diff=B-A)

#--------------------------------------------------------------------------

# calculate all other mean differences (as per above)

#--------------------------------------------------------------------------

MD_all <- data %>% gather(key='sched', value='phase', 5:(4+PermissibleNo))

MD_all <- MD_all %>% group_by(id, sched, phase) %>%

summarise(score=mean(score, na.rm=TRUE)) %>%

spread(key=phase, value=score) %>%

mutate(diff2=B-A)

#--------------------------------------------------------------------------

# compare actual MD to all MD for each participant (as per above)

#--------------------------------------------------------------------------

MD_all <- inner_join(MD_all, MD_actual, by='id') #join data together

MD_all <- MD_all %>% mutate(n=(mean_diff)>=(diff2)) #mark true or false if actual MD equal to or greater than the hypothetical one

pvals <- MD_all %>% ungroup() %>% group_by(id) %>% summarise(pval=(PermissibleNo+1-sum(n))/PermissibleNo) #turn this into a pvalue

#--------------------------------------------------------------------------

# Pool pvalues (as per above)

#--------------------------------------------------------------------------

mySumz <- sumz(pvals$pval) #Stouffer's Z from the metap package

#--------------------------------------------------------------------------

# Effect size (as per above)

#--------------------------------------------------------------------------

ES <-effect_size_MB(data$score, data$phase, data$id, data$session) # calculates between-case effect size

#save the resulting Z and p values for this imputation and move on to the next one

Results$p[k] <- as.numeric(mySumz$p)

Results$z[k] <- mySumz$z

Results$d[k] <- ES$delta_hat # d statistic (Adjusted for small samples)

Results$d_V[k] <- ES$V_delta_hat # variance of d

}

# 4. Once all imputations have been run, pool results using Rubin's rules

z <- mean(Results$z) #average z score across k imputations #

pvalue <- 2*pnorm(abs(z), lower.tail = F) #p value assuming 2 tailed distribution

d <- mean(Results$d)

d_V <- mean(Results$d_V)
